# Supplementary material for: Protocol describing a systematic review and mixed methods consensus process to define the deteriorated ward patient
Source: BMJ Open. 2022 Sep 19;12(9):e057614. doi: 10.1136/bmjopen-2021-057614 (PMC9486195; doi:10.1136/bmjopen-2021-057614)
Supplement: Author's manuscript [file bmjopen-2021-057614.draft_revisions.pdf]

BMJ Open is committed to open peer review. As part of this commitment we make the peer review history of every article we publish publicly available.

When an article is published we post the peer reviewers' comments and the authors' responses online. We also post the versions of the paper that were used during peer review. These are the versions that the peer review comments apply to.

The versions of the paper that follow are the versions that were submitted during the peer review process. They are not the versions of record or the final published versions. They should not be cited or distributed as the published version of this manuscript.

BMJ Open is an open access journal and the full, final, typeset and author-corrected version of record of the manuscript is available on our site with no access controls, subscription charges or pay-per-view fees (http://bmjopen.bmj.com).

If you have any questions on BMJ Open's open peer review process please email info.bmjopen@bmj.com

BMJ Open

ESTABLISHING CONSENSUS DEFINITIONS FOR THE DETERIORATED WARD PATIENT – A PROTOCOL

Journal:	*BMJ Open*
Manuscript ID	bmjopen-2021-057614
Article Type:	Protocol
Date Submitted by the Author:	23-Sep-2021

Complete List of Authors:	Malycha, James; The University of Adelaide Faculty of Health and Medical Sciences, Department of Acute Care Medicine;<br>Andersen, Chris; The George Institute for Global Health<br>Redfern, Oliver; Oxford University, Nuffield Department of Clinical Neurosciences;<br>Ludbrook, Guy; The University of Adelaide<br>Watkinson, Peter; University of Oxford, Kadoorie Centre for Critical Care research and Education<br>Young, Duncan; University of Oxford, Nuffield Department of Clinical Neurosciences<br>Peake, Sandra; The University of Adelaide<br>Subbe, Christian; Bangor University, School of Medical Sciences<br>Dykes, L; Flinders University<br>Phillips, Adam; University of South Australia<br>Flabouris, Arthas; The University of Adelaide, Acute Care Medicine<br>Jones, Daryl; Austin Health, Intensive Care Unit Austin Hospital

Keywords:	Adult intensive & critical care < INTENSIVE & CRITICAL CARE, Health & safety < HEALTH SERVICES ADMINISTRATION & MANAGEMENT, Quality in health care < HEALTH SERVICES ADMINISTRATION & MANAGEMENT, Risk management < HEALTH SERVICES ADMINISTRATION & MANAGEMENT

SCHOLARONE™
Manuscripts

ESTABLISHING CONSENSUS DEFINITIONS FOR THE DETERIORATED WARD PATIENT – A PROTOCOL

Authors

Dr James Malycha (corresponding author) MBBS FCICM
Staff Specialist, Intensive Care, The Queen Elizabeth Hospital
Email: James.Malycha@ndcn.ox.ac.uk

Dr Chris Andersen (joint first author) BSc (Hon1) BA MBBS (Hon) MClinTRes FCICM
Staff Specialist, Intensive Care, Royal North Shore Hospital
Clinical Lecturer and PhD Candidate, Sydney University
Research Fellow, The George Institute for Global Health

Dr Oliver Redfern MBBS PhD
Post-Doctoral Research Fellow, Nuffield Department of Clinical Neurosciences, University of Oxford

Professor Sandra Peake BM BS BSc (Hons) FCICM Ph D
Director Intensive Care, The Queen Elizabeth Hospital
Professor, Faculty of Health and Medical Sciences, University of Adelaide

Dr Chris Subbe MBBS FRACP
Consultant Acute, Respiratory & Critical Care Medicine, Ysbyty Gwynedd, Bangor
Senior Clinical Lecturer, Bangor University, Bangor

Mr Lukah Dykes BSc
Data Fellow, South Australia Health and Medical Research Institute
Associate Lecturer, College of Medicine and Public Health, Flinders University

Mr Adam Phillips BSc BPharm (Hons) CHIA
Health Informatics, Central Adelaide Local Health Network
Adjunct Research Fellow, Clinical and Health Sciences, University of South Australia

Professor Guy Ludbrook PhD MBBS FANZCA
Staff Specialist, Anaesthesia, Royal Adelaide Hospital
Professor of Anaesthesia, Faculty of Health and Medical Science, University of Adelaide

Professor Duncan J. Young MD DM FRCA FMedSci
Professor of Intensive Care Medicine, Nuffield Department of Clinical Neurosciences, University of Oxford

For peer review only

Professor Peter J. Watkinson MD ChB MRCP FDICM
Staff Specialist, Intensive Care, Oxford University Hospitals Trust
Professor of Intensive Care Medicine, Nuffield Department of Clinical Neurosciences, University of Oxford

A/Prof Arthas Flabouris MBBS MD FCICM FANZCA FACAsM PGDipAvMed PGDipEcho
Staff Specialist, Intensive Care, Royal Adelaide Hospital
Clinical Associate Professor, Faculty of Health and Medical Sciences, University of Adelaide

A/Prof Daryl Jones BSc(Hons) MB BS FRACP FCICM MD PhD
Staff Specialist, Intensive Care, Austin Hospital
Adjunct Associate Professor, University Melbourne
Adjunct Senior Research Fellow, Department of Epidemiology and Preventative Medicine, Monash University

Key words: Critical Care, Rapid Response System, Patient Deterioration, Outcome Measures, Consensus.

Word count: 1344 words

For peer review only

Abstract

Introduction

Most patients admitted to hospital recover with treatments that can be administered on the general ward. However, a small cohort deteriorate to the extent that they require augmented organ support. In observational studies evaluating this cohort, proxy outcomes are used, including unplanned transfer from general ward to the Intensive Care Unit (ICU), cardiac arrest and death. However, these outcomes measures have limitations. This protocol aims to describe a method to better define the deteriorated ward patient. To achieve this, we will use a literature review and validated consensus building methods.

Methods and Analysis

1. We will undertake a systematic literature review to identify existing definitions.
2. An international modified Delphi study will generate a 'short list' of candidate definitions.
3. A Nominal Group Technique (NGT) meeting, informed by the data generated from the first two stages, will be used to complete the consensus building process.

The results of the study will be made available to international researchers. It is anticipated the definitions will then be evaluated and iterated by different research teams. These results will inform the international research community on the relevance of the definitions and their potential usefulness. Ideally, the definitions will hasten the development and improve the performance of automated, Electronic Medical Record (EMR) linked, digital models that accurately predict which general ward patients will require augmented organ support (as opposed to predicting death, cardiac arrest or unplanned ICU admission).

Ethics and Dissemination

Ethics approval will not be required for this study. Results generated from this study will be disseminated through publication and presentation at national and international scientific meetings.

For peer review only

Strengths and Limitations of this study

- The study described in this protocol will generate a novel outcome measure that could enhance research into predictive analytics used for patients who deteriorate on the general ward
- The methods described to generate consensus are well validated
- The methods described may be deployed in parallel fields of clinical research to good effect
- The results of the study described in this protocol have the potential to improve the care of a very large patient cohort (i.e., general ward patients at risk of clinical deterioration)
- The pandemic limits the ability of study participants to meet in person (although advances in online conference technology mitigates this to some degree)

For peer review only

Introduction

Most patients admitted to hospital recover with treatments that can be administered on the general ward. However, a small cohort within this population deteriorate to the extent that they require augmented organ support **(Figure 1).** (1) In observational studies evaluating this cohort, proxy outcomes are most often used. These include unplanned transfer from the general ward to the Intensive Care Unit (ICU), cardiac arrest and death. (3, 4) However, the decision to transfer patients to the ICU is dependent on multiple factors, including personalised advance care directives, clinician opinion, local care escalation protocols such as Early Warning Score (EWS) systems, and the availability of ICU resources. (5) These factors introduce subjectivity and variability when ICU admission is chosen as an outcome measure, hindering evaluation of interventions designed to improve the care of these patients. (6–11)

Cardiac arrest and death are well-defined and easily measured but are often a very late marker of deterioration. Additionally, cardiac arrest frequency is rare, which limits its use for derivation and validation processes, even in large patient data sets. We aim to define this deteriorated ward patient cohort more accurately, using the time-point of when the need for augmented organ support first occurs. We will use validated consensus methods to generate the definitions using a diverse international panel of stakeholders.

Aim and Objectives

This protocol aims to describe a method to better define the deteriorated ward patient. To achieve this, we will use a literature review and validated consensus building methods.

Methods

Consensus definitions will be established in three stages. Firstly, we will undertake a systematic literature review to identify existing definitions for clinical deterioration (12) Secondly, an international modified Delphi study will generate a 'short list' of candidate definitions. Finally, a Nominal Group Technique (NGT) meeting, informed by the data generated from the first two stages, will be used to complete the consensus building process. Definitions are expected to be organ system specific and will not be designed as real-time adjuncts to clinical decision making. Both Delphi and NGT are validated methods for establishing consensus in health care settings. (13–15)

For peer review only - http://bmjopen.bmj.com/site/about/guidelines.xhtml

Stage 1 - Literature Review

The Preferred Reporting of Observational Studies and Meta-Analysis (PRISMA) guidelines will be used to conduct a literature review on current definitions for the deteriorating ward patient.(12) Data sources will include MEDLINE, EMBASE, CINAHL and CENTRAL (for full names see Abbreviations section). Additional papers will be included from the references of review articles. An example of the search criteria is included in the **Table 1**

Table 1.

Step	Term	Studies
1	INTENSIVE CARE UNITS/	49151
2	CRITICAL CARE/st	3674
3	("intensive care" or ICU* or ITU*).ti.	46127
4	1 or 2 or 3	74307
5	PATIENT ADMISSION/st 891	891
6	TRIAGE/st 1205	1205
7	PATIENT SELECTION/ 60959	60959
8	(admission* or admit* or access* or triage*).ti. 96353	96353
9	5 or 6 or 7 or 8 157554	157554
10	4 and 9 4318	4318
11	(Criteria or assessment or optim* or survey or decision* or evaluat* or consensus or standard* or measure* or algorithm* or tool* or instrument* or guideline* or framework* or method* or strateg*).ti.	2114207
12	10 and 11	555

Table 1. Example search criteria using MEDLINE

Stage 2 - Delphi Study

Participants

We aim to include 60 participants in the Delphi study. Participants will be recruited through the International Society for Rapid Response Systems, the International Forum for Acute Care Trialists and relevant national societies. No formal inclusion criteria will be used however potential participants will be considered based on relevant clinical and research experience,

For peer review only - http://bmjopen.bmj.com/site/about/guidelines.xhtml

with the aim of ensuring participants are representative of eventual end-users. These will include hospital-based clinical staff working in regional, rural and metropolitan hospitals as well as non-clinician content experts such as researchers and digital health specialists.

Patient and Public Involvement

A small number of health consumer representatives will also be recruited to participate in

Round 1 – Establishing initial definitions (time frame: two months)

Results of the literature review and a list of potential domains, variables and/or parameters will be distributed via email to participants. Participants will provide structured feedback on the merits (or otherwise) of each item. These will then be coalesced into an initial list of potential definitions. Any missed items will be submitted to the process for consideration.

Round 2 – Ranking potential definitions (time frame: two months)

Participants will rank each potential definition using a 9-point Likert System. Consensus will be defined as 70% of respondents classifying the definitions as 'critical' (score of 7 - 9) and less than 15% determining the definition to be 'not relevant' (score 1 - 3). The results will be aggregated. Any criteria achieving a score of > 70% 'not relevant' will be removed.

R*ound 3 – Refining aggregated results (time frame: two months)*

Aggregated results will be presented to each participant. Definitions that remain, but that have not yet achieved consensus, will be rescored. These results will then be aggregated, and the list finalised. Any definitions that have not achieved consensus after three rounds of scoring will be excluded.

Round 4 – Generating thresholds (time frame: two months)

Participants will propose one threshold for each organ specific definition with an evidence-based justification for the threshold.

Stage 3 - Nominal Group Technique/Consensus meeting (time frame: 1 day)

Nominal Group Technique (NGT) is a validated method for establishing consensus on a specific issue or range of related issues. (14) The NGT meeting will aim to include a diverse range of clinical stakeholders. The target number of participants will be 15 - 20.

For peer review only - http://bmjopen.bmj.com/site/about/guidelines.xhtml

Participants

Participants (both professional and public) will be selected and invited using the same process as described for the Delphi. Participants need not have been involved in the first two stages of the study to take part.

A trained facilitator will lead NGT participants through the structured multi-stage process: Firstly, participants will be presented with an overview of the NGT meeting rationale and aim. Next, participants will be presented with the results of the systematic review and the Delphi process. Participants will then spend 10 - 15 minutes writing a list of bullet point reflections and opinions on the definitions provided, including an opportunity to advocate for additional relevant data not previously included. The facilitator will then get participants to list one reflection/opinion that is yet to be presented. Each original point will be transcribed onto a screen or whiteboard, so all participants can consider and review. This may take several rounds until opinions are exhausted (the aim being to enable all participants to express their views and prevent specific participants having a disproportionate influence).

Participants will then place each definition into two columns: one for inclusion and one for exclusion. The results of this activity will be tabulated and presented. Consensus will be confirmed if more than 70% of participants support its inclusion or exclusion. (16) If there is a lack of consensus on a definition, then the contentious item will be taken back to the group for reappraisal and repeat voting until either consensus or stalemate (two additional voting rounds without consensus) is reached.

The final stage of the NGT will determine the thresholds (if required) for each of the definitions. Participants will write down opinions/reflections on potential thresholds and these will be collated with each original perspective transcribed. Participants will then provide specific thresholds for relevant definitions; these results will be tabulated, and discussion will be encouraged. The facilitators will present numerous potential thresholds based on the feedback and these will again be voted on. The final set of definition thresholds will be presented to the group and pending agreement, recorded for subsequent publication.

Dissemination

For peer review only - http://bmjopen.bmj.com/site/about/guidelines.xhtml

Results generated from this study will be disseminated through publication and presentation at national and international scientific meetings.

Discussion

In this protocol, we have described a method using international expert consensus to define the deteriorated ward patient as the time-point that the need for augmented organ support first occurs. To our knowledge this is the first study to undertake this research task. This research represents an important step in improving the precision of outcome measures used for the development and evaluation of future clinical deterioration prediction models. The proposed work is challenging. It aims to use consensus building methods that are current best practice. The development of the definitions will be an iterative process.

Once published, the results of the study will be made available to international researchers. It is anticipated the definitions will then be evaluated and iterated in observational studies by different research teams. These results will inform the international research community on the relevance of the definitions and their potential usefulness. Ideally, the definitions will hasten the development and improve the performance of automated, EMR linked, digital models that accurately predict which general ward patients will require augmented organ support (as opposed to predicting death, cardiac arrest or unplanned ICU admission).

Trial Status

This is Protocol Version 1 dated 23/08/2021. Recruitment for this study has not begun. It is expected that recruitment for participation in the Delphi and NGT will be completed by Jan 31st, 2022.

Abbreviations

MEDLINE – Medical Literature Analysis and Retrieval System Online
CINAHL – Cumulative Index to Nursing and Allied Health Literature
EMBASE – Excerpta Medica database
CENTRAL – Cochrane Index to Nursing and Allied Health Literature and the Cochrane Central Register of controlled trials

For peer review only - http://bmjopen.bmj.com/site/about/guidelines.xhtml

Declarations

Acknowledgments

JM would like the acknowledge the University of Adelaide, Department of Acute Medicine, who are administering his PhD.

Contributors

JM and CA designed the study. JM, CA and DJ undertook the methodological planning. JM and CA wrote the manuscript. All authors commented on successive drafts and approved the final manuscript.

Funding

PW and OR are both supported by the National Institute for Health and Research Biomedical Research Centre, Oxford. CS does consultancy work for Philips Healthcare.

Data and material

All data and material generated from the study will be published.

Competing interests

None

Patient consent for publication

N/A

Ethics approval

Ethics approval will not be required for this study.

Provenance and peer review

Open access

For peer review only

References

1. Orosz J, Bailey M, Udy A, et al.: Unplanned ICU Admission from Hospital Wards after Rapid Response Team Review in Australia and New Zealand. *Crit Care Med* 2020; 48:E550–E556
2. Blanch L, Abillama FF, Amin P, et al.: Triage decisions for ICU admission: Report from the Task Force of the World Federation of Societies of Intensive and Critical Care Medicine. *J Crit Care* 2016; 36:301–305
3. Smith MEB, Chiovaro JC, O'Neil M, et al.: Early Warning System Scores for Clinical Deterioration in Hospitalized Patients: A Systematic Review. *Ann Am Thorac Soc* 2014; 11:1454–1465
4. Alam N, Hobbelink EL, van Tienhoven AJ, et al.: The impact of the use of the Early Warning Score (EWS) on patient outcomes: A systematic review. *Resuscitation* 2014; 85:587–594
5. Nunnally M, Fowler CS, Bailey H, et al.: ICU Admission, Discharge, and Triage Guidelines. 2016.
6. Churpek MM, Yuen TC, Edelson DP: Predicting clinical deterioration in the hospital: The impact of outcome selection. *Resuscitation* 2013; 84:564–568
7. Churpek MM, Carey KA, Dela Merced N, et al.: Validation of Early Warning Scores at Two Long-Term Acute Care Hospitals. *Crit Care Med* 2019; 47:e962–e965
8. Kipnis P, Turk BJ, Wulf DA, et al.: Development and validation of an electronic medical record-based alert score for detection of inpatient deterioration outside the ICU. *J Biomed Inform* 2016; 64:10–19
9. O'Brien C, Goldstein BA, Shen Y, et al.: Development, Implementation, and Evaluation of an In-Hospital Optimized Early Warning Score for Patient Deterioration. *MDM Policy Pract* 2020; 5:1–10
10. Escobar GJ, Laguardia JC, Turk BJ, et al.: Early detection of impending physiologic deterioration among patients who are not in intensive care: Development of predictive models using data from an automated electronic medical record. *J Hosp Med* 2012; 7:388–395
11. Alvarez CA, Clark CA, Zhang S, et al.: Predicting out of intensive care unit cardiopulmonary arrest or death using electronic medical record data. *BMC Med Inform Decis Mak* 2013; 13:28
12. Moher D, Liberati A, Tetzlaff J, et al.: Systematic Reviews and Meta-Analyses: The

For peer review only - http://bmjopen.bmj.com/site/about/guidelines.xhtml

PRISMA Statement. *Annu Intern Med* 2009; 151:264–269

13. Sinha IP, Smyth RL, Williamson PR: Using the Delphi Technique to Determine Which Outcomes to Measure in Clinical Trials: Recommendations for the Future Based on a Systematic Review of Existing Studies. *PLoS Med* 2011; 8:e1000393

14. Grech C: Use of consensus development to establish national research priorities in critical care. *Aust Crit Care* 2000; 13:163

15. Jones J, Hunter D: Qualitative Research: Consensus methods for medical and health services research. *Bmj* 1995; 311:376

16. Rankin NM, McGregor D, Butow PN, et al.: Adapting the nominal group technique for priority setting of evidence-practice gaps in implementation science. *BMC Med Res Methodol* 2016; 16:1–9

For peer review only

For peer review only

For peer review only - http://bmjopen.bmj.com/site/about/guidelines.xhtml

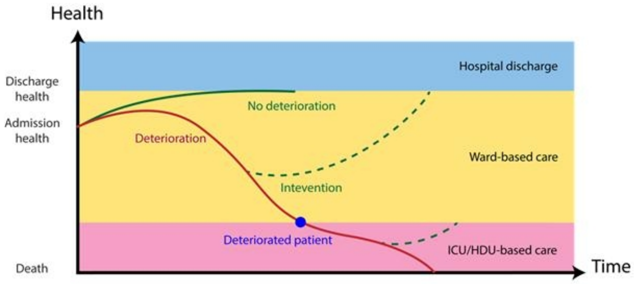

Figure 1.

A schematic representation of potential trajectories for hospitalised patient. Most patients are expected to progress along the green line. However, in a small cohort significant deterioration will occur. This may be subject to early intervention or will reach an end point at which they are no longer suitable for management in a ward environment and will be determined to have 'deteriorated'.

338x254mm (72 x 72 DPI)

Government of South Australia
SA Health

Research Checklist

Item	Page number
Author Information	1, 2
Manuscript length and formatting	2
Tables	Supplementary material
Figures	Uploaded as 'image'
References	10, 11
Supplementary Files	Uploaded
Statements	9
Acknowledgements	9
Suggested reviewers	None
Research reporting checklists	5 (PRISMA)
Reproducing figures	Original and need no permissions

For peer review only

BMJ Open

A Protocol Describing A Systematic Review And Mixed Methods Consensus Process To Define The Deteriorated Ward Patient

Journal:	*BMJ Open*
Manuscript ID	bmjopen-2021-057614.R1
Article Type:	Protocol
Date Submitted by the Author:	21-Feb-2022
Complete List of Authors:	Malycha, James; The University of Adelaide Faculty of Health and Medical Sciences, Department of Acute Care Medicine;<br>Andersen, Chris; The George Institute for Global Health<br>Redfern, Oliver; Oxford University, Nuffield Department of Clinical Neurosciences;<br>Peake, Sandra; The University of Adelaide<br>Subbe, Christian; Bangor University, School of Medical Sciences<br>Dykes, L; Flinders University<br>Phillips, Adam; University of South Australia<br>Ludbrook, Guy; The University of Adelaide<br>Young, Duncan; University of Oxford, Nuffield Department of Clinical Neurosciences<br>Watkinson, Peter; University of Oxford, Kadoorie Centre for Critical Care research and Education<br>Flabouris, Arthas; The University of Adelaide, Acute Care Medicine<br>Jones, Daryl; Austin Health, Intensive Care Unit Austin Hospital
**Primary Subject Heading**:	Intensive care
Secondary Subject Heading:	Health informatics, Intensive care, Research methods
Keywords:	Adult intensive & critical care < INTENSIVE & CRITICAL CARE, Health & safety < HEALTH SERVICES ADMINISTRATION & MANAGEMENT, Quality in health care < HEALTH SERVICES ADMINISTRATION & MANAGEMENT, Risk management < HEALTH SERVICES ADMINISTRATION & MANAGEMENT, Health informatics < BIOTECHNOLOGY & BIOINFORMATICS

SCHOLARONE™
Manuscripts

A Protocol Describing A Systematic Review And Mixed Methods Consensus Process To Define The Deteriorated Ward Patient

Dr James Malycha (corresponding author) MBBS FCICM PhD

Staff Specialist, Intensive Care, The Queen Elizabeth Hospital

Email: James.Malycha@ndcn.ox.ac.uk

Dr Chris Andersen (joint first author) BSc (Hon1) BA MBBS (Hon) MClinTRes FCICM

Staff Specialist, Intensive Care, Royal North Shore Hospital

Clinical Lecturer and PhD Candidate, Sydney University

Research Fellow, The George Institute for Global Health

Dr Oliver Redfern MBBS PhD

Post-Doctoral Research Fellow, Nuffield Department of Clinical Neurosciences, University of Oxford

Professor Sandra Peake BM BS BSc (Hons) FCICM Ph D

Director Intensive Care, The Queen Elizabeth Hospital

Professor, Faculty of Health and Medical Sciences, University of Adelaide

Dr Chris Subbe MBBS FRACP

Consultant Acute, Respiratory & Critical Care Medicine, Ysbyty Gwynedd, Bangor

For peer review only

Senior Clinical Lecturer, Bangor University, Bangor

Mr Lukah Dykes BSc

Data Fellow, South Australia Health and Medical Research Institute

Associate Lecturer, College of Medicine and Public Health, Flinders University

Mr Adam Phillips BSc BPharm (Hons) CHIA

Health Informatics, Central Adelaide Local Health Network

Adjunct Research Fellow, Clinical and Health Sciences**,** University of South Australia

Professor Guy Ludbrook PhD MBBS FANZCA

Staff Specialist, Anaesthesia, Royal Adelaide Hospital

Professor of Anaesthesia, Faculty of Health and Medical Science, University of Adelaide

Professor Duncan J. Young MD DM FRCA FMedSci

Professor of Intensive Care Medicine, Nuffield Department of Clinical Neurosciences, University of Oxford

Professor Peter J. Watkinson MD ChB MRCP FDICM

Staff Specialist, Intensive Care, Oxford University Hospitals Trust

Professor of Intensive Care Medicine, Nuffield Department of Clinical Neurosciences, University of Oxford

For peer review only

A/Prof Arthas Flabouris MBBS MD FCICM FANZCA FACAsM PGDipAvMed PGDipEcho

Staff Specialist, Intensive Care, Royal Adelaide Hospital

Clinical Associate Professor, Faculty of Health and Medical Sciences, University of Adelaide

A/Prof Daryl Jones BSc(Hons) MB BS FRACP FCICM MD PhD

Staff Specialist, Intensive Care, Austin Hospital

Adjunct Associate Professor, University Melbourne

Adjunct Senior Research Fellow, Department of Epidemiology and Preventative Medicine, Monash University

For peer review only

Key words: Critical Care, Rapid Response System, Deteriorated Patient, Deterioration, Outcome Measures, Consensus.

Word count: words 2090

Abstract

Introduction

Most patients admitted to hospital recover with treatments that can be administered on the general ward. However, a small but important group deteriorate to the extent that they require augmented organ support. In observational studies evaluating this cohort, proxy outcomes are used. These include unplanned transfer from the general ward to the Intensive Care Unit (ICU), cardiac arrest and death. These outcome measures introduce subjectivity and variability, which in turn hinders the development and accuracy of the increasing numbers of Electronic Medical Record (EMR) linked, digital tools designed to predict clinical Deterioration.

Methods and Analysis

We will undertake a systematic literature review to identify existing definitions. The aim of the review will be to identify existing generic, syndrome specific and organ specific definitions for clinically deteriorated, hospitalised adult patients. An international modified Delphi study will generate a short list of candidate definitions. We aim to include 60 participants in the Delphi study which is consistent with previous Delphi surveys in critical care outcomes and should be adequate to achieve a suitably diverse international sample of stakeholders. A Nominal Group Technique (NGT) meeting, informed by the data generated from the first two stages, will be used to complete the consensus building process for a final list of definitions. Nominal Group Technique (NGT) is a validated method for establishing consensus on a specific issue or range of related issues that uses a trained facilitator to take a group of participants through a structured process. The NGT meeting will aim to include a diverse range of clinical stakeholders. The target number of participants will be 15 - 20.

For peer review only - http://bmjopen.bmj.com/site/about/guidelines.xhtml

Ethics and Dissemination

Ethical approval for the Delphi survey and nominal group technique meeting will be sought via the Northern Sydney Human Research Ethics Committee. Results generated from this study will be disseminated through publication and presentation at national and international scientific meetings.

Strengths and Limitations of this study

The specific research question and the methods are novel. The systematic review will be thorough and will ensure all relevant available published data will inform the subsequent modified Delphi survey and nominal group technique, which are the most common and well validated methods for establishing consensus in the medical literature. The definition(s) generated by the study will be evaluated for use as outcome measures when developing predictive tools for clinical deterioration. Determining when to implement augmented organ support varies between individual clinicians and is influenced by institutional resources and health care settings. It is anticipated bringing the multiple opinions and experiences together into one set of definitions will be challenging.

For peer review only

Introduction

Most patients admitted to hospital recover with treatments that can be administered on the general ward. However, a small but important group deteriorate to the extent that they require augmented organ support **(Figure 1).** [1] In observational studies evaluating this cohort proxy outcomes are used. These include unplanned transfer from the general ward to the Intensive Care Unit (ICU), cardiac arrest and death. [2] The decision to transfer patients to the ICU is dependent on multiple factors, including personalised advance care directives, clinician opinion, local care escalation protocols such as Early Warning Score (EWS) systems, and the availability of ICU resources. [3] Cardiac arrest and death are well-defined and easily measured but are often a very late marker of deterioration. Additionally, cardiac arrest frequency is rare, which limits its use for derivation and validation processes, even in large patient data sets. These factors introduce subjectivity and variability to research that uses these as outcome measures, which in turn hinders the development and accuracy of the increasing numbers of Electronic Medical Record (EMR) linked, algorithmic tools designed to predict clinical Deterioration. [4,5]

Aims

The primary aim of this study is to establish a consensus definition (or set of syndrome or organ specific definitions) for the deteriorated ward patient. The secondary aim is to do so using data that is commonly available in most EMR's. The definitions will target the time-point that the requirement for augmented organ support first occurs (whilst taking into consideration contextual variables like advanced care directives).

For peer review only

Methods

Consensus definitions will be established in three stages. Firstly, a systematic literature review will be undertaken to identify existing generic, syndrome and organ specific definitions of clinical deterioration. Secondly, an international modified Delphi study will generate a short list of candidate definitions. Finally, a Nominal Group Technique (NGT) meeting, informed by the data generated from the first two stages, will generate the final definition(s).

Stage 1 - Literature Review

Objective

To identify existing generic, syndrome specific and organ specific definitions for clinically deteriorated, hospitalised, adult patients.

Methods

This systematic review will follow the requirements of Preferred Reporting Items for Systematic Reviews and Meta-analyses Protocol (PRISMA-P). [6]

Phenomenon of interest

Studies that characterise or define deteriorated ward patients. These may be regarding generic (or generalised) deteriorated states associated with the traditional early warning score systems or novel algorithmic, automated deteriorated patient surveillance tools. [7] They may be regarding syndromes specific to clinical deterioration, such as sepsis and associated definitions including Sepsis-3. [8] They may also be regarding specific organ dysfunctions, acute decompensated liver failure being a relevant and common example.

For peer review only

For peer review only - http://bmjopen.bmj.com/site/about/guidelines.xhtml

Search Strategy

Studies will be identified using Medical Literature Analysis and Retrieval System Online (MEDLINE), Excerpta Medica database (EMBASE), Cumulative Index to Nursing and Allied Health Literature (CINAHL), the Cochrane Database of Systematic Reviews and the Cochrane Central Register of Controlled Trials (CENTRAL). Additional papers will be sourced from references of included studies, reviews articles and studies from the author libraries.

Search Terms

The following search terms will be included: intensive care, critical care, critical, emergency, deteriorating, deteriorated, definition, electronic patient record, electronic health record, electronic patient record, predictive, unplanned intensive care unit admission, adverse event, terminology, nomenclature, acute, acute care, severe, sudden, rapid response, early warning score, sepsis, septic, shock, shocked, hypoxia, COVID-19, respiratory failure, cardiac failure, liver failure, renal failure, anuria, hypotension, instability, unstable, threshold, acute organ dysfunction and criteria. Additional search terms will be considered after initial trials with the above search terms. A trained medical librarian from the Central Adelaide Local Health Network will assist with the search process.

Study Selection and Data Extraction

Two unblinded researchers will independently screen the titles and abstracts of identified studies against the inclusion and exclusion criteria. Disagreement and/or uncertainty regarding study eligibility will be resolved using a third party. Both researchers will independently extract data from included studies using DistillerSR (Evidence Partners, Ottawa, Canada) which will also be used to manage data and identify duplicates.

For peer review only - http://bmjopen.bmj.com/site/about/guidelines.xhtml

Inclusion criteria

Quantitative or qualitative studies published in peer reviewed journals describing definitions of, or criteria specific to, adult (defined as > 16 years of age) clinical deterioration will be eligible for inclusion in this review. Studies published from January 2000 until the day of search completion will be included and no language restrictions will be applied. Google Translate will be used for non-English studies.

Exclusion criteria

Case studies, editorials, grey-literature, letters, practice guidelines and abstract-only reports will be excluded.

Quality Assessment

Different tools will be used to assess the quality of included studies, depending on the type of study. For outcome measure studies the COnsensus-based Standards for the selection of health Measurement INstruments (COSMIN) Risk of Bias checklist will be used. [9] For prediction model studies, the Prediction model Risk Of Bias ASsessment Tool (PROBAST) will be used. [10] For qualitative studies the Joanna Briggs Institute Evidence Based Practice Checklist will be used. [11] For clinical guidelines the AGREE II tool will be used. [12] For Randomised Controlled Trials the Cochrane Collaboration Risk of Bias 2 guidelines will be used. [13]

Data Synthesis and analysis

We will generate a list of organ specific, syndrome specific and generic definitions of the deteriorated patient from included studies. These data will be presented in table and text form.

For peer review only - http://bmjopen.bmj.com/site/about/guidelines.xhtml

Stage 2 - Delphi Study

Participants

We aim to include 60 participants in the Delphi study which is consistent with previous Delphi surveys in critical care outcomes and should be adequate to achieve a suitably diverse international sample of stakeholders. [14] Participants will be recruited through the International Society for Rapid Response Systems, the International Forum for Acute Care Trialists and relevant national societies. No formal inclusion criteria will be used however potential participants will be considered based on relevant clinical and research experience, with the aim of ensuring participants are representative of eventual end-users. These will include hospital-based clinical staff working in regional, rural and metropolitan hospitals as well as non-clinician content experts such as researchers and digital health specialists.

Patient and Public Involvement

A small number of health consumer representatives will also be recruited to participate.

Round 1 – Establishing initial definitions (time frame: two months)

Results of the literature review and a list of potential domains, variables and/or parameters will be distributed via email to participants. Participants will provide structured feedback on the merits (or otherwise) of each item. These will then be coalesced into an initial list of potential definitions. Any missed items will be submitted to the process for consideration.

Round 2 – Ranking potential definitions (time frame: two months)

For peer review only - http://bmjopen.bmj.com/site/about/guidelines.xhtml

Participants will rank each potential definition using a 9-point Likert System that is recommended by the Grading of Recommendations, Assessment, Development and Evaluation (GRADE) Working Group Handbook for evaluating outcomes measures. Based on previous work in outcomes research we have defined consensus as 70% of respondents classifying the definitions as 'critical' (score of 7 - 9) and less than 15% determining the definition to be 'not relevant' (score 1 - 3). The results will be aggregated. Any criteria achieving a score of > 70% 'not relevant' will be removed.

Round 3 – Refining aggregated results (time frame: two months)

Aggregated results will be presented to each participant. Definitions that remain, but that have not yet achieved consensus, will be rescored. These results will then be aggregated, and the list finalised. Any definitions that have not achieved consensus after three rounds of scoring will be excluded.

Round 4 – Generating thresholds (time frame: two months)

Participants will propose one threshold for each organ specific definition with an evidence-based justification for the threshold.

Stage 3 - Nominal Group Technique/Consensus meeting (time frame: 1 day)

Nominal Group Technique (NGT) is a validated method for establishing consensus on a specific issue or range of related issues that uses a trained facilitator to take a group of participants through a structured process. [15][16] The NGT meeting will aim to include a diverse range of clinical stakeholders. The target number of participants will be 15 - 20.

For peer review only - http://bmjopen.bmj.com/site/about/guidelines.xhtml

Participants

Participants (both professional and public) will be selected and invited using the same process as described for the Delphi. Participants need not have been involved in the first two stages of the study to take part.

A trained facilitator will lead NGT participants through the structured multi-stage process: Firstly, participants will be presented with an overview of the NGT meeting rationale and aim. Next, participants will be presented with the results of the systematic review and the Delphi process. Participants will then spend 10 - 15 minutes writing a list of bullet point reflections and opinions on the definitions provided, including an opportunity to advocate for additional relevant data not previously included. The facilitator will then get participants to list one reflection/opinion that is yet to be presented. Each original point will be transcribed onto a screen or whiteboard, so all participants can consider and review. This may take several rounds until opinions are exhausted (the aim being to enable all participants to express their views and prevent specific participants having a disproportionate influence).

Participants will then place each definition into two columns: one for inclusion and one for exclusion. The results of this activity will be tabulated and presented. Consensus will be confirmed if more than 70% of participants support its inclusion or exclusion. [16] If there is a lack of consensus on a definition, then the contentious item will be taken back to the group for reappraisal and repeated voting until either consensus or stalemate (two additional voting rounds without consensus) is reached.

For peer review only - http://bmjopen.bmj.com/site/about/guidelines.xhtml

The final stage of the NGT will determine the thresholds (if required) for each of the definitions. Participants will write down opinions/reflections on potential thresholds and these will be collated with each original perspective transcribed. Participants will then provide specific thresholds for relevant definitions; these results will be tabulated, and discussion will be encouraged. The facilitators will present numerous potential thresholds based on the feedback and these will again be voted on. The final set of definition thresholds will be presented to the group and pending agreement, recorded for subsequent publication.

Dissemination

Results generated from this study will be disseminated through publication and presentation at national and international scientific meetings.

Discussion

This protocol describes a three step consensus building process for developing a definition (or set of definitions) for the deteriorated ward patient. The purpose of this research is to aid the development and improve the performance of automated, EMR linked, digital models that predict clinical deterioration in general ward patients. It will also be useful in evaluations of Early Warning Score and Rapid Response Systems. It is important to note the definition(s) will not be designed as real time decision making adjuncts or to replace complex clinical decision making.

The work has a number of weaknesses. The pandemic has limited the ability of those involved to gather in person (which is the most effective way to build consensus). The increased familiarity with virtual meeting platforms mitigates this to a certain degree. Determining when to implement augmented organ support varies between individual clinicians and is influenced by institutional

For peer review only - http://bmjopen.bmj.com/site/about/guidelines.xhtml

resources and health care settings. Indeed, defining 'augmented organ support' is itself fraught with difficulty. It is anticipated bringing the multiple opinions and experiences together into one set of definitions is going to be challenging. Additionally, maintaining consistency across generic, organ specific (i.e., respiratory failure) and syndrome specific (i.e., sepsis) definitions of the deteriorated patient will require discipline and careful planning or risk being of little use in research or clinical practice.

This work has a number of strengths. The specific research question and the methods are novel. The systematic review will be thorough and will ensure all relevant available published data will inform the subsequent modified Delphi survey and nominal group technique, which are the most common and well validated methods for establishing consensus in the medical literature. The definition(s) generated by the study will be evaluated for use as outcome measures when developing predictive tools for clinical deterioration. These may in turn reduce the dependence on the traditional outcome measures, including death, cardiac arrest or unplanned ICU admission, which have specific shortcomings that hinder performance. The definitions will be derived from commonly available EMR data, making them widely applicable as digital health care systems become more widespread. There may be additional uses of the consensus definition(s) beyond the remit of this study, such as comparing acuity between different health care providers and guiding policy. Overall, the published results will (through various means) be relevant to the many thousands of patients annually who clinically deteriorate on hospital wards.

For peer review only - http://bmjopen.bmj.com/site/about/guidelines.xhtml

Trial Status

This is Protocol Version 1 dated 11/02/2022. Recruitment for this study has not begun. It is expected that recruitment for participation in the Delphi and NGT will be completed by July 31st, 2022.

Legend

Figure 1. The schematic representation of potential trajectories for hospitalised patients. Most patients progress along the green line. However, in a small cohort significant deterioration will occur. This may be subject to early intervention or will reach an end point at which they are no longer suitable for management in a ward environment and will be defined a 'deteriorated'

Declarations

Acknowledgments

Nil

Contributors

JM and CA conceptualised the study. JM, CA, OR, CS and DJ undertook the initial methodological planning. GL, PW, DY, SP, AF undertook secondary analysis of the methods and provided updates and corrections. AP and LD provided technical advice. JM and CA wrote the initial manuscript. OR, GL, PW, DY, SP, CS, LD, AP, AF and DJ edited successive drafts and approved the final manuscript.

For peer review only - http://bmjopen.bmj.com/site/about/guidelines.xhtml

Funding

PW and OR are both supported by the National Institute for Health and Research Biomedical Research Centre, Oxford. CS does consultancy work for Philips Healthcare.

Data and material

All data and material generated from the study will be published.

Competing interests

None

Patient consent for publication

N/A

Ethics approval

Ethical approval for the Delphi survey and nominal group technique meeting will be sought via the Northern Sydney Human Research Ethics Committee.

Provenance and peer review

Open access

For peer review only - http://bmjopen.bmj.com/site/about/guidelines.xhtml

References

1 Orosz J, Bailey M, Udy A, *et al.* Unplanned ICU Admission from Hospital Wards after Rapid Response Team Review in Australia and New Zealand. *Crit Care Med* 2020;**48**:E550–6.

2 Churpek MM, Yuen TC, Edelson DP. Predicting clinical deterioration in the hospital: The impact of outcome selection. *Resuscitation* 2013;**84**:564–8.

3 Malycha J, Redfern O, Pimentel M, *et al.* Evaluation of a digital system to predict unplanned admissions to the intensive care unit: A mixed-methods approach. *Resusc Plus* 2022;**9**:100193.

4 Escobar GJ, Liu VX, Schuler A, *et al.* Automated Identification of Adults at Risk for In-Hospital Clinical Deterioration. *N Engl J Med* 2020;**383**:1951–60.

5 Churpek MM, Yuen TC, Edelson DP. Risk stratification of hospitalized patients on the wards. *Chest* 2013;**143**:1758–65.

6 Moher D, Liberati A, Tetzlaff J, *et al.* Reprint—Preferred Reporting Items for Systematic Reviews and Meta-Analyses: The PRISMA Statement. 2017;**89**:873–80.

7 Mann KD, Good NM, Fatehi F, *et al.* Predicting Patient Deterioration: A Review of Tools in the Digital Hospital Setting. *J Med Internet Res* 2021;**23**:e28209.

8 Singer M, Deutschman CS, Seymour CW, *et al.* The Third International Consensus Definitions for Sepsis and Septic Shock (Sepsis-3). *JAMA* 2016;**315**:801–10.

9 Mokkink LB, de Vet HCW, Prinsen CAC, *et al.* COSMIN Risk of Bias checklist for systematic reviews of Patient-Reported Outcome Measures. *Qual Life Res* 2018;**27**:1171–9.

10 Wolff RF, Moons KGM, Riley RD, *et al.* PROBAST: A Tool to Assess the Risk of Bias and Applicability of Prediction Model Studies. *Ann Intern Med* 2019;**170**:51–8.

11 Vardell E, Malloy M. Joanna Briggs Institute: an evidence-based practice database. *Med Ref Serv Q* 2013;**32**:434–42.

12 AGREE Collaboration. Development and validation of an international appraisal instrument for assessing the quality of clinical practice guidelines: the AGREE project. *Qual Saf Health Care* 2003;**12**:18–23.

13 Collaborative C. An introduction to Risk of Bias 2. Cochrane. https://methods.cochrane.org/risk-bias-2

14 Sinha IP, Smyth RL, Williamson PR. Using the Delphi technique to determine which outcomes to measure in clinical trials: Recommendations for the future based on a systematic review of existing studies. *PLoS Med* 2011;**8**:e1000393.

For peer review only - http://bmjopen.bmj.com/site/about/guidelines.xhtml

15 Grech C. Use of consensus development to establish national research priorities in critical care. *Aust Crit Care* 2000;**13**:163.

16 Rankin NM, McGregor D, Butow PN, *et al.* Adapting the nominal group technique for priority setting of evidence-practice gaps in implementation science. *BMC Med Res Methodol* 2016;**16**:1–9.

For peer review only

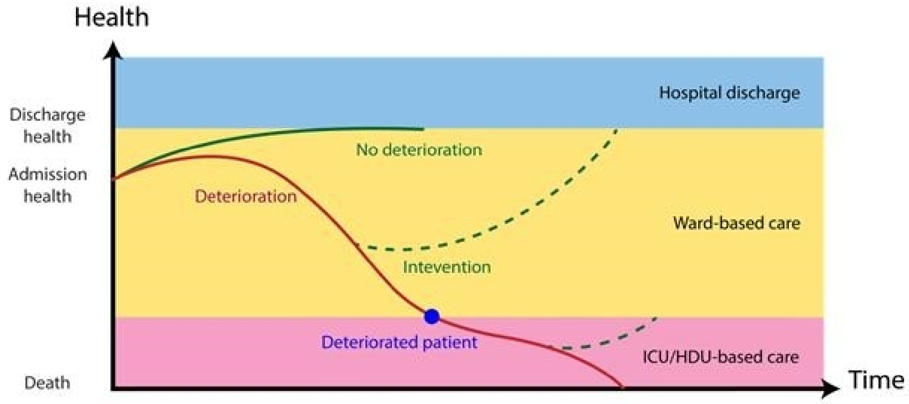

Figure 1. A schematic representation of potential trajectories for hospitalised patients. Most patients progress along the green line. However, in a small cohort, significant deterioration will occur. This deterioration may be subject to early intervention or will reach an end point at which time the patient is no longer suitable for management in a ward environment and will be defined as 'deteriorated'

177x80mm (96 x 96 DPI)

Government of South Australia
SA Health

Research Checklist

Item	Page number
Author Information	1, 2
Manuscript length and formatting	2
Tables	Supplementary material
Figures	Uploaded as 'image'
References	10, 11
Supplementary Files	Uploaded
Statements	9
Acknowledgements	9
Suggested reviewers	None
Research reporting checklists	5 (PRISMA)
Reproducing figures	Original and need no permissions

For peer review only

BMJ Open

A Protocol Describing A Systematic Review And Mixed Methods Consensus Process To Define The Deteriorated Ward Patient

Journal:	*BMJ Open*
Manuscript ID	bmjopen-2021-057614.R2
Article Type:	Protocol
Date Submitted by the Author:	11-Apr-2022
Complete List of Authors:	Malycha, James; The University of Adelaide Faculty of Health and Medical Sciences, Department of Acute Care Medicine;<br>Andersen, Chris; The George Institute for Global Health<br>Redfern, Oliver; Oxford University, Nuffield Department of Clinical Neurosciences;<br>Peake, Sandra; The University of Adelaide<br>Subbe, Christian; Bangor University, School of Medical Sciences<br>Dykes, L; Flinders University<br>Phillips, Adam; University of South Australia<br>Ludbrook, Guy; The University of Adelaide<br>Young, Duncan; University of Oxford, Nuffield Department of Clinical Neurosciences<br>Watkinson, Peter; University of Oxford, Kadoorie Centre for Critical Care research and Education<br>Flabouris, Arthas; The University of Adelaide, Acute Care Medicine<br>Jones, Daryl; Austin Health, Intensive Care Unit Austin Hospital
**Primary Subject Heading**:	Intensive care
Secondary Subject Heading:	Health informatics, Intensive care, Research methods
Keywords:	Adult intensive & critical care < INTENSIVE & CRITICAL CARE, Health & safety < HEALTH SERVICES ADMINISTRATION & MANAGEMENT, Quality in health care < HEALTH SERVICES ADMINISTRATION & MANAGEMENT, Risk management < HEALTH SERVICES ADMINISTRATION & MANAGEMENT, Health informatics < BIOTECHNOLOGY & BIOINFORMATICS

SCHOLARONE™
Manuscripts

A Protocol Describing A Systematic Review And Mixed Methods Consensus Process To Define The Deteriorated Ward Patient

Dr James Malycha (corresponding author) MBBS FCICM PhD

Staff Specialist, Intensive Care, The Queen Elizabeth Hospital

Email: James.Malycha@ndcn.ox.ac.uk

Dr Chris Andersen (joint first author) BSc (Hon1) BA MBBS (Hon) MClinTRes FCICM

Staff Specialist, Intensive Care, Royal North Shore Hospital

Clinical Lecturer and PhD Candidate, Sydney University

Research Fellow, The George Institute for Global Health

Dr Oliver Redfern MBBS PhD

Post-Doctoral Research Fellow, Nuffield Department of Clinical Neurosciences, University of Oxford

Professor Sandra Peake BM BS BSc (Hons) FCICM Ph D

Director Intensive Care, The Queen Elizabeth Hospital

Professor, Faculty of Health and Medical Sciences, University of Adelaide

Dr Chris Subbe MBBS FRACP

Consultant Acute, Respiratory & Critical Care Medicine, Ysbyty Gwynedd, Bangor

For peer review only

Senior Clinical Lecturer, Bangor University, Bangor

Mr Lukah Dykes BSc

Data Fellow, South Australia Health and Medical Research Institute

Associate Lecturer, College of Medicine and Public Health, Flinders University

Mr Adam Phillips BSc BPharm (Hons) CHIA

Health Informatics, Central Adelaide Local Health Network

Adjunct Research Fellow, Clinical and Health Sciences, University of South Australia

Professor Guy Ludbrook PhD MBBS FANZCA

Staff Specialist, Anaesthesia, Royal Adelaide Hospital

Professor of Anaesthesia, Faculty of Health and Medical Science, University of Adelaide

Professor Duncan J. Young MD DM FRCA FMedSci

Professor of Intensive Care Medicine, Nuffield Department of Clinical Neurosciences, University of Oxford

Professor Peter J. Watkinson MD ChB MRCP FDICM

Staff Specialist, Intensive Care, Oxford University Hospitals Trust

Professor of Intensive Care Medicine, Nuffield Department of Clinical Neurosciences, University of Oxford

For peer review only

A/Prof Arthas Flabouris MBBS MD FCICM FANZCA FACAsM PGDipAvMed PGDipEcho

Staff Specialist, Intensive Care, Royal Adelaide Hospital

Clinical Associate Professor, Faculty of Health and Medical Sciences, University of Adelaide

A/Prof Daryl Jones BSc(Hons) MB BS FRACP FCICM MD PhD

Staff Specialist, Intensive Care, Austin Hospital

Adjunct Associate Professor, University Melbourne

Adjunct Senior Research Fellow, Department of Epidemiology and Preventative Medicine, Monash University

For peer review only

Key words: Critical Care, Rapid Response System, Deteriorated Patient, Deterioration, Outcome Measures, Consensus.

Word count: words 2090

For peer review only - http://bmjopen.bmj.com/site/about/guidelines.xhtml

Abstract

Introduction

Most patients admitted to hospital recover with treatments that can be administered on the general ward. A small but important group deteriorate however, and require augmented organ support in areas with increased nursing to patient ratios. In observational studies evaluating this cohort, proxy outcomes such as unplanned Intensive Care Unit (ICU) admission, cardiac arrest and death are used. These outcome measures introduce subjectivity and variability, which in turn hinders the development and accuracy of the increasing numbers of Electronic Medical Record (EMR) linked digital tools designed to predict clinical deterioration. Here we describe a protocol for developing a new outcome measure using mixed methods to address these limitations.

Methods and Analysis

We will undertake a systematic literature review to identify existing generic, syndrome specific and organ specific definitions for clinically deteriorated, hospitalised adult patients. An international modified Delphi study will generate a short list of candidate definitions. A Nominal Group Technique (NGT), using a trained facilitator, will then take a diverse group of stakeholders through a structured process. The NGT process will be informed by the data generated from the first two stages and will achieve final consensus of a definition for the deteriorated ward patient that is readily extractable from the major EMR platforms.

Ethics and Dissemination

Ethical approval for the Delphi survey and nominal group technique meeting are currently being sought via the Central Adelaide Local Health Network Human Research Ethics Committee.

For peer review only - http://bmjopen.bmj.com/site/about/guidelines.xhtml

Results generated from this study will be disseminated through publication and presentation at national and international scientific meetings.

Strengths and Limitations of this study

- This work addresses an important knowledge gap and will assist in developing predictive tools for clinical deterioration.
- The systematic review will be thorough and will scope all relevant available published data to inform the development of the definitions
- The international consensus process will include patients, researchers and clinicians from across different health settings improving the definitions validity
- Determining when to implement augmented organ support varies between individual clinicians and health care settings and bringing the multiple opinions and experiences together into one set of definitions will be challenging.
- The final definition(s) will need to be extractable from the EMR to serve their purpose and will require ongoing refinement and evaluation in large international data sets.

For peer review only

For peer review only - http://bmjopen.bmj.com/site/about/guidelines.xhtml

Introduction

Most patients admitted to hospital recover with treatments that can be administered on the general ward. A small but important group deteriorate however, to the extent that they require augmented organ support **(Figure 1).** [1] In observational studies evaluating this cohort proxy outcomes are used. These include unplanned transfer from the general ward to the Intensive Care Unit (ICU), cardiac arrest and death. [2] The decision to transfer patients to the ICU is dependent on multiple factors, including personalised advance care directives, clinician opinion, local care escalation protocols such as Early Warning Score (EWS) systems, and the availability of ICU resources. [3] Cardiac arrest and death are well-defined and easily measured but are often a very late marker of deterioration. Additionally, cardiac arrest frequency is rare, which limits its use for derivation and validation processes, even in large patient data sets. These factors introduce subjectivity and variability to research that uses these as outcome measures, which in turn hinders the development and accuracy of the increasing numbers of Electronic Medical Record (EMR) linked, algorithmic tools designed to predict clinical Deterioration. [4,5]

Aims

The primary aim of this study is to establish an international consensus definition (or set of syndrome or organ specific definitions) for the deteriorated ward patient. The secondary aim is to do so using data that is commonly available in most EMR's. The definitions will target the time-point that the requirement for augmented organ support first occurs (whilst taking into consideration contextual variables like advanced care directives).

For peer review only - http://bmjopen.bmj.com/site/about/guidelines.xhtml

Methods

Consensus definitions will be established in three stages. Firstly, a systematic literature review will be undertaken to identify existing generic, syndrome and organ specific definitions of clinical deterioration. Secondly, an international modified Delphi study will generate a short list of candidate definitions. Finally, a Nominal Group Technique (NGT) meeting, informed by the data generated from the first two stages, will generate the final definition(s).

Stage 1 - Literature Review

Objective

To identify existing generic, syndrome specific and organ specific definitions for clinically deteriorated, hospitalised, adult patients.

Methods

This systematic review will follow the requirements of Preferred Reporting Items for Systematic Reviews and Meta-analyses Protocol (PRISMA-P). [6]

Phenomenon of interest

Studies that characterise or define deteriorated ward patients. These may be regarding generic (or generalised) deteriorated states associated with the traditional early warning score systems or novel algorithmic, automated deteriorated patient surveillance tools. [7] They may be regarding syndromes specific to clinical deterioration, such as sepsis and associated definitions including Sepsis-3. [8] They may also be regarding specific organ dysfunctions, acute decompensated liver failure being a relevant and common example.

For peer review only

For peer review only - http://bmjopen.bmj.com/site/about/guidelines.xhtml

Search Strategy

Studies will be identified using Medical Literature Analysis and Retrieval System Online (MEDLINE), Excerpta Medica database (EMBASE), Cumulative Index to Nursing and Allied Health Literature (CINAHL), the Cochrane Database of Systematic Reviews and the Cochrane Central Register of Controlled Trials (CENTRAL). Additional papers will be sourced from references of included studies, reviews articles and studies from the author libraries.

Search Terms

The following search terms will be included: intensive care, critical care, critical, emergency, deteriorating, deteriorated, definition, electronic patient record, electronic health record, electronic patient record, predictive, unplanned intensive care unit admission, adverse event, terminology, nomenclature, acute, acute care, severe, sudden, rapid response, early warning score, sepsis, septic, shock, shocked, hypoxia, COVID-19, respiratory failure, cardiac failure, liver failure, renal failure, anuria, hypotension, instability, unstable, threshold, acute organ dysfunction and criteria. Additional search terms will be considered after initial trials with the above search terms. A trained medical librarian from the Central Adelaide Local Health Network will assist with the search process.

Study Selection and Data Extraction

Two researchers will independently screen the titles and abstracts of identified studies against the inclusion and exclusion criteria. Disagreement and/or uncertainty regarding study eligibility will be resolved using a third party. Both researchers will independently extract data from included studies using DistillerSR (Evidence Partners, Ottawa, Canada) which will also be used to manage data and identify duplicates.

For peer review only - http://bmjopen.bmj.com/site/about/guidelines.xhtml

Inclusion criteria

Quantitative or qualitative studies published in peer reviewed journals describing definitions of, or criteria specific to, adult (defined as > 16 years of age) clinical deterioration will be eligible for inclusion in this review. Studies published from January 2000 until the day of search completion will be included and no language restrictions will be applied. Google Translate will be used for non-English studies.

Exclusion criteria

Case studies, editorials, grey-literature, letters, practice guidelines and abstract-only reports will be excluded.

Quality Assessment

Different tools will be used to assess the quality of included studies, depending on the type of study. For outcome measure studies the COnsensus-based Standards for the selection of health Measurement INstruments (COSMIN) Risk of Bias checklist will be used. [9] For prediction model studies, the Prediction model Risk Of Bias Assessment Tool (PROBAST) will be used. [10] For qualitative studies the Joanna Briggs Institute Evidence Based Practice Checklist will be used. [11] For clinical guidelines the AGREE II tool will be used. [12] For Randomised Controlled Trials the Cochrane Collaboration Risk of Bias 2 guidelines will be used. [13]

Data Synthesis and analysis

We will generate a list of organ specific, syndrome specific and generic definitions of the deteriorated patient from included studies. These data will be presented in table and text form.

For peer review only - http://bmjopen.bmj.com/site/about/guidelines.xhtml

Stage 2 - Delphi Study

Participants

We aim to include 60 participants in the Delphi study which is consistent with previous Delphi surveys in critical care outcomes and should be adequate to achieve a suitably diverse international sample of stakeholders. [14] Participants will be recruited through the International Society for Rapid Response Systems, the International Forum for Acute Care Trialists and relevant national societies. No formal inclusion criteria will be used however potential participants will be considered based on relevant clinical and research experience, with the aim of ensuring participants are representative of eventual end-users. These will include hospital-based clinical staff working in regional, rural and metropolitan hospitals as well as non-clinician content experts such as researchers and digital health specialists.

Patient and Public Involvement

A small number of health consumer representatives will also be recruited to participate.

Round 1 – Establishing initial definitions (time frame: two months)

Results of the literature review and a list of potential domains, variables and/or parameters will be distributed via email to participants. Participants will provide structured feedback on the merits (or otherwise) of each item. These will then be coalesced into an initial list of potential definitions. Any missed items will be submitted to the process for consideration.

Round 2 – Ranking potential definitions (time frame: two months)

For peer review only - http://bmjopen.bmj.com/site/about/guidelines.xhtml

Participants will rank each potential definition using a 9-point Likert System that is recommended by the Grading of Recommendations, Assessment, Development and Evaluation (GRADE) Working Group Handbook for evaluating outcomes measures. Based on previous work in outcomes research we have defined consensus as 70% of respondents classifying the definitions as ‘critical’ (score of 7 - 9) and less than 15% determining the definition to be ‘not relevant’ (score 1 - 3). The results will be aggregated. Any criteria achieving a score of > 70% ‘not relevant’ will be removed.

Round 3 – Refining aggregated results (time frame: two months)

Aggregated results will be presented to each participant. Definitions that remain, but that have not yet achieved consensus, will be rescored. These results will then be aggregated, and the list finalised. Any definitions that have not achieved consensus after three rounds of scoring will be excluded.

Round 4 – Generating thresholds (time frame: two months)

Participants will propose one threshold for each organ specific definition with an evidence-based justification for the threshold.

Stage 3 - Nominal Group Technique/Consensus meeting (time frame: 1 day)

Nominal Group Technique (NGT) is a validated method for establishing consensus on a specific issue or range of related issues that uses a trained facilitator to take a group of participants through a structured process. [15][16] The NGT meeting will aim to include a diverse range of clinical stakeholders. The target number of participants will be 15 - 20.

For peer review only - http://bmjopen.bmj.com/site/about/guidelines.xhtml

Participants

Participants (both professional and public) will be selected and invited using the same process as described for the Delphi. Participants need not have been involved in the first two stages of the study to take part.

A trained facilitator will lead NGT participants through the structured multi-stage process: Firstly, participants will be presented with an overview of the NGT meeting rationale and aim. Next, participants will be presented with the results of the systematic review and the Delphi process. Participants will then spend 10 - 15 minutes writing a list of bullet point reflections and opinions on the definitions provided, including an opportunity to advocate for additional relevant data not previously included. The facilitator will then get participants to list one reflection/opinion that is yet to be presented. Each original point will be transcribed onto a screen or whiteboard, so all participants can consider and review. This may take several rounds until opinions are exhausted (the aim being to enable all participants to express their views and prevent specific participants having a disproportionate influence).

Participants will then place each definition into two columns: one for inclusion and one for exclusion. The results of this activity will be tabulated and presented. Consensus will be confirmed if more than 70% of participants support its inclusion or exclusion. [16] If there is a lack of consensus on a definition, then the contentious item will be taken back to the group for reappraisal and repeated voting until either consensus or stalemate (two additional voting rounds without consensus) is reached.

For peer review only - http://bmjopen.bmj.com/site/about/guidelines.xhtml

The final stage of the NGT will determine the thresholds (if required) for each of the definitions. Participants will write down opinions/reflections on potential thresholds and these will be collated with each original perspective transcribed. Participants will then provide specific thresholds for relevant definitions; these results will be tabulated, and discussion will be encouraged. The facilitators will present numerous potential thresholds based on the feedback and these will again be voted on. The final set of definition thresholds will be presented to the group and pending agreement, recorded for subsequent publication.

Dissemination

Results generated from this study will be disseminated through publication and presentation at national and international scientific meetings.

Discussion

This protocol describes a three-step consensus building process for developing a definition (or set of definitions) for the deteriorated ward patient. The purpose of this research is to aid the development and improve the performance of automated, EMR linked, digital models that predict clinical deterioration in general ward patients. It will also be useful in evaluations of Early Warning Score and Rapid Response Systems. It is important to note the definition(s) will not be designed as real time decision making adjuncts or to replace complex clinical decision making.

The work has a number of weaknesses. The pandemic has limited the ability of those involved to gather in person (which is the most effective way to build consensus). The increased familiarity with virtual meeting platforms mitigates this to a certain degree. Determining when to implement augmented organ support varies between individual clinicians and is influenced by institutional

For peer review only - http://bmjopen.bmj.com/site/about/guidelines.xhtml

resources and health care settings. Indeed, defining ‘augmented organ support’ is itself fraught with difficulty. It is anticipated bringing the multiple opinions and experiences together into one set of definitions is going to be challenging. Additionally, maintaining consistency across generic, organ specific (i.e., respiratory failure) and syndrome specific (i.e., sepsis) definitions of the deteriorated patient will require discipline and careful planning or risk being of little use in research or clinical practice.

This work has a number of strengths. The specific research question and the methods are novel. The systematic review will be thorough and will ensure all relevant available published data will inform the subsequent modified Delphi survey and nominal group technique, which are the most common and well validated methods for establishing consensus in the medical literature. The definition(s) generated by the study will be evaluated for use as outcome measures when developing predictive tools for clinical deterioration. These may in turn reduce the dependence on the traditional outcome measures, including death, cardiac arrest or unplanned ICU admission, which have specific shortcomings that hinder performance. The definitions will be derived from commonly available EMR data, making them widely applicable as digital health care systems become more widespread. There may be additional uses of the consensus definition(s) beyond the remit of this study, such as comparing acuity between different health care providers and guiding policy. Overall, the published results will (through various means) be relevant to the many thousands of patients annually who clinically deteriorate on hospital wards.

For peer review only

Trial Status

This is Protocol Version 1 dated 11/02/2022. Recruitment for this study has not begun. It is expected that recruitment for participation in the Delphi and NGT will be completed by July 31st, 2022.

Legend

Figure 1. The schematic representation of potential trajectories for hospitalised patients. Most patients progress along the green line. However, in a small cohort significant deterioration will occur. This may be subject to early intervention or will reach an end point at which they are no longer suitable for management in a ward environment and will be defined a 'deteriorated'

Declarations

Acknowledgments

Nil

Contributors

JM and CA conceptualised the study. JM, CA, OR, CS and DJ undertook the initial methodological planning. GL, PW, DY, SP, AF undertook secondary analysis of the methods and provided updates and corrections. AP and LD provided technical advice. JM and CA wrote the initial manuscript. OR, GL, PW, DY, SP, CS, LD, AP, AF and DJ edited successive drafts and approved the final manuscript.

For peer review only

For peer review only - http://bmjopen.bmj.com/site/about/guidelines.xhtml

Funding

PW and OR are both supported by the National Institute for Health and Research Biomedical Research Centre, Oxford. CS does consultancy work for Philips Healthcare.

Data and material

All data and material generated from the study will be published.

Competing interests

None

Patient consent for publication

N/A

Ethics approval

Ethical approval for the Delphi survey and nominal group technique meeting is currently being reviewed by the Central Adelaide Local Health Network Research and Ethics Committee.

Provenance and peer review

Open access

For peer review only

References

1 Orosz J, Bailey M, Udy A, *et al.* Unplanned ICU Admission from Hospital Wards after Rapid Response Team Review in Australia and New Zealand. *Crit Care Med* 2020;**48**:E550–6.

2 Churpek MM, Yuen TC, Edelson DP. Predicting clinical deterioration in the hospital: The impact of outcome selection. *Resuscitation* 2013;**84**:564–8.

3 Malycha J, Redfern O, Pimentel M, *et al.* Evaluation of a digital system to predict unplanned admissions to the intensive care unit: A mixed-methods approach. *Resusc Plus* 2022;**9**:100193.

4 Escobar GJ, Liu VX, Schuler A, *et al.* Automated Identification of Adults at Risk for In-Hospital Clinical Deterioration. *N Engl J Med* 2020;**383**:1951–60.

5 Churpek MM, Yuen TC, Edelson DP. Risk stratification of hospitalized patients on the wards. *Chest* 2013;**143**:1758–65.

6 Moher D, Liberati A, Tetzlaff J, *et al.* Reprint—Preferred Reporting Items for Systematic Reviews and Meta-Analyses: The PRISMA Statement. 2017;**89**:873–80.

7 Mann KD, Good NM, Fatehi F, *et al.* Predicting Patient Deterioration: A Review of Tools in the Digital Hospital Setting. *J Med Internet Res* 2021;**23**:e28209.

8 Singer M, Deutschman CS, Seymour CW, *et al.* The Third International Consensus Definitions for Sepsis and Septic Shock (Sepsis-3). *JAMA* 2016;**315**:801–10.

9 Mokkink LB, de Vet HCW, Prinsen CAC, *et al.* COSMIN Risk of Bias for systematic reviews of Patient-Reported Outcome Measures. *Qual Life Res* 2018;**27**:1171–9.

10 Wolff RF, Moons KGM, Riley RD, *et al.* PROBAST: A Tool to Assess the Risk of Bias and Applicability of Prediction Model Studies. *Ann Intern Med* 2019;**170**:51–8.

11 Vardell E, Malloy M. Joanna Briggs Institute: an evidence-based practice database. *Med Ref Serv Q* 2013;**32**:434–42.

12 AGREE Collaboration. Development and validation of an international appraisal instrument for assessing the quality of clinical practice guidelines: the AGREE project. *Qual Saf Health Care* 2003;**12**:18–23.

13 Collaborative C. An introduction to Risk of Bias 2. Cochrane. https://methods.cochrane.org/risk-bias-2

14 Sinha IP, Smyth RL, Williamson PR. Using the Delphi technique to determine which outcomes to measure in clinical trials: Recommendations for the future based on a systematic review of existing studies. *PLoS Med* 2011;**8**:e1000393.

For peer review only

For peer review only - http://bmjopen.bmj.com/site/about/guidelines.xhtml

15 Grech C. Use of consensus development to establish national research priorities in critical care. *Aust Crit Care* 2000;**13**:163.

16 Rankin NM, McGregor D, Butow PN, *et al.* Adapting the nominal group technique for priority setting of evidence-practice gaps in implementation science. *BMC Med Res Methodol* 2016;**16**:1–9.

For peer review only

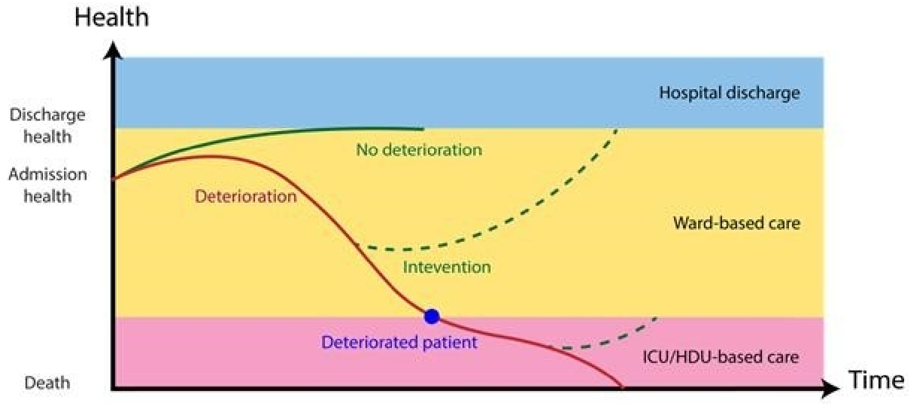

Figure 1. A schematic representation of potential trajectories for hospitalised patients. Most patients progress along the green line. However, in a small cohort, significant deterioration will occur. This deterioration may be subject to early intervention or will reach an end point at which time the patient is no longer suitable for management in a ward environment and will be defined as 'deteriorated'

177x80mm (96 x 96 DPI)

PRISMA-P (Preferred Reporting Items for Systematic review and Meta-Analysis Protocols) 2015 checklist: recommended items to address in a systematic review protocol*

Section and topic	Item No	Checklist item	Comments
**ADMINISTRATIVE INFORMATION**			
Title:			
Identification	1a	Identify the report as a protocol of a systematic review	Yes
Update	1b	If the protocol is for an update of a previous systematic review, identify as such	N/A
Registration	2	If registered, provide the name of the registry (such as PROSPERO) and registration number	Not registered
Authors:			
Contact	3a	Provide name, institutional affiliation, e-mail address of all protocol authors; provide physical mailing address of corresponding author	Yes
Contributions	3b	Describe contributions of protocol authors and identify the guarantor of the review	Yes
Amendments	4	If the protocol represents an amendment of a previously completed or published protocol, identify as such and list changes; otherwise, state plan for documenting important protocol amendments	N/A
Support:			
Sources	5a	Indicate sources of financial or other support for the review	Yes
Sponsor	5b	Provide name for the review funder and/or sponsor	Yes
Role of sponsor or funder	5c	Describe roles of funder(s), sponsor(s), and/or institution(s), if any, in developing the protocol	Yes
**INTRODUCTION**			
Rationale	6	Describe the rationale for the review in the context of what is already known	Yes
Objectives	7	Provide an explicit statement of the question(s) the review will address with reference to participants, interventions, comparators, and outcomes (PICO)	Yes
**METHODS**			
Eligibility criteria	8	Specify the study characteristics (such as PICO, study design, setting, time frame) and report characteristics (such as years considered, language, publication status) to be used as criteria for eligibility for the review	Yes
Information sources	9	Describe all intended information sources (such as electronic databases, contact with study authors, trial registers or other grey literature sources) with planned dates of coverage	Yes
Search strategy	10	Present draft of search strategy to be used for at least one electronic database, including planned limits, such that it could be repeated	Yes
Study records:			
Data management	11a	Describe the mechanism(s) that will be used to manage records and data throughout the review	Yes
Selection process	11b	State the process that will be used for selecting studies (such as two independent reviewers) through each phase of the	Yes

For peer review only

		review (that is, screening, eligibility and inclusion in meta-analysis)	
Data collection process	11c	Describe planned method of extracting data from reports (such as piloting forms, done independently, in duplicate), any processes for obtaining and confirming data from investigators	Yes
Data items	12	List and define all variables for which data will be sought (such as PICO items, funding sources), any pre-planned data assumptions and simplifications	Yes
Outcomes and prioritization	13	List and define all outcomes for which data will be sought, including prioritization of main and additional outcomes, with rationale	N/A
Risk of bias in individual studies	14	Describe anticipated methods for assessing risk of bias of individual studies, including whether this will be done at the outcome or study level, or both; state how this information will be used in data synthesis	Yes
Data synthesis	15a	Describe criteria under which study data will be quantitatively synthesised	N/A
	15b	If data are appropriate for quantitative synthesis, describe planned summary measures, methods of handling data and methods of combining data from studies, including any planned exploration of consistency (such as I^2, Kendall's τ)	N/A
	15c	Describe any proposed additional analyses (such as sensitivity or subgroup analyses, meta-regression)	N/A
	15d	If quantitative synthesis is not appropriate, describe the type of summary planned	Yes
Meta-bias(es)	16	Specify any planned assessment of meta-bias(es) (such as publication bias across studies, selective reporting within studies)	N/A
Confidence in cumulative evidence	17	Describe how the strength of the body of evidence will be assessed (such as GRADE)	N/A

*** It is strongly recommended that this checklist be read in conjunction with the PRISMA-P Explanation and Elaboration (cite when available) for important clarification on the items. Amendments to a review protocol should be tracked and dated. The copyright for PRISMA-P (including checklist) is held by the PRISMA-P Group and is distributed under a Creative Commons Attribution Licence 4.0.**

From: Shamseer L, Moher D, Clarke M, Ghersi D, Liberati A, Petticrew M, Shekelle P, Stewart L, PRISMA-P Group. Preferred reporting items for systematic review and meta-analysis protocols (PRISMA-P) 2015: elaboration and explanation. BMJ. 2015 Jan 2;349(jan02 1):g7647.

For peer review only

BMJ Open

A Protocol Describing A Systematic Review And Mixed Methods Consensus Process To Define The Deteriorated Ward Patient

Journal:	*BMJ Open*
Manuscript ID	bmjopen-2021-057614.R3
Article Type:	Protocol
Date Submitted by the Author:	23-Aug-2022
Complete List of Authors:	Malycha, James; The Queen Elizabeth Hospital, Intensive Care; The University of Adelaide, Department of Acute Care Medicine<br>Andersen, Chris; The George Institute for Global Health<br>Redfern, Oliver; Oxford University, Nuffield Department of Clinical Neurosciences;<br>Peake, Sandra; The University of Adelaide<br>Subbe, Christian; Bangor University, School of Medical Sciences<br>Dykes, L; Flinders University<br>Phillips, Adam; University of South Australia<br>Ludbrook, Guy; The University of Adelaide<br>Young, Duncan; University of Oxford, Nuffield Department of Clinical Neurosciences<br>Watkinson, Peter; University of Oxford, Kadoorie Centre for Critical Care research and Education<br>Flabouris, Arthas; The University of Adelaide, Acute Care Medicine<br>Jones, Daryl; Austin Health, Intensive Care Unit Austin Hospital
**Primary Subject Heading**:	Intensive care
Secondary Subject Heading:	Health informatics, Intensive care, Research methods
Keywords:	Adult intensive & critical care < INTENSIVE & CRITICAL CARE, Health & safety < HEALTH SERVICES ADMINISTRATION & MANAGEMENT, Quality in health care < HEALTH SERVICES ADMINISTRATION & MANAGEMENT, Risk management < HEALTH SERVICES ADMINISTRATION & MANAGEMENT, Health informatics < BIOTECHNOLOGY & BIOINFORMATICS

SCHOLARONE™
Manuscripts

A Protocol Describing A Systematic Review And Mixed Methods Consensus Process To Define The Deteriorated Ward Patient

Dr James Malycha (corresponding author) MBBS FCICM PhD

Staff Specialist, Intensive Care, The Queen Elizabeth Hospital

Senior Clinical Lecturer, Department of Acute Care Medicine, University of Adelaide

Email: James.Malycha@sa.gov.au

Dr Chris Andersen (joint first author) BSc (Hon1) BA MBBS (Hon) MClinTRes FCICM

Staff Specialist, Intensive Care, Royal North Shore Hospital

Clinical Lecturer and PhD Candidate, Sydney University

Research Fellow, The George Institute for Global Health

Dr Oliver Redfern MBBS PhD

Post-Doctoral Research Fellow, Nuffield Department of Clinical Neurosciences, University of Oxford

Professor Sandra Peake BM BS BSc (Hons) FCICM Ph D

Director Intensive Care, The Queen Elizabeth Hospital

Professor, Faculty of Health and Medical Sciences, University of Adelaide

For peer review only

Dr Chris Subbe MBBS FRACP

Consultant Acute, Respiratory & Critical Care Medicine, Ysbyty Gwynedd, Bangor

Senior Clinical Lecturer, Bangor University, Bangor

Mr Lukah Dykes BSc

Data Fellow, South Australia Health and Medical Research Institute

Associate Lecturer, College of Medicine and Public Health, Flinders University

Mr Adam Phillips BSc BPharm (Hons) CHIA

Health Informatics, Central Adelaide Local Health Network

Adjunct Research Fellow, Clinical and Health Sciences, University of South Australia

Professor Guy Ludbrook PhD MBBS FANZCA

Staff Specialist, Anaesthesia, Royal Adelaide Hospital

Professor of Anaesthesia, Faculty of Health and Medical Science, University of Adelaide

Professor Duncan J. Young MD DM FRCA FMedSci

Professor of Intensive Care Medicine, Nuffield Department of Clinical Neurosciences, University of Oxford

Professor Peter J. Watkinson MD ChB MRCP FDICM

Staff Specialist, Intensive Care, Oxford University Hospitals Trust

Professor of Intensive Care Medicine, Nuffield Department of Clinical Neurosciences, University of Oxford

For peer review only

A/Prof Arthas Flabouris MBBS MD FCICM FANZCA FACAsM PGDipAvMed PGDipEcho

Staff Specialist, Intensive Care, Royal Adelaide Hospital

Clinical Associate Professor, Faculty of Health and Medical Sciences, University of Adelaide

A/Prof Daryl Jones BSc(Hons) MB BS FRACP FCICM MD PhD

Staff Specialist, Intensive Care, Austin Hospital

Adjunct Associate Professor, University Melbourne

Adjunct Senior Research Fellow, Department of Epidemiology and Preventative Medicine, Monash University

For peer review only

Key words: Critical Care, Rapid Response System, Deteriorated Patient, Deterioration, Outcome Measures, Consensus.

Word count: words 2090

For peer review only - http://bmjopen.bmj.com/site/about/guidelines.xhtml

Abstract

Introduction

Most patients admitted to hospital recover with treatments that can be administered on the general ward. A small but important group deteriorate however and require augmented organ support in areas with increased nursing to patient ratios. In observational studies evaluating this cohort, proxy outcomes such as unplanned Intensive Care Unit (ICU) admission, cardiac arrest and death are used. These outcome measures introduce subjectivity and variability, which in turn hinders the development and accuracy of the increasing numbers of Electronic Medical Record (EMR) linked digital tools designed to predict clinical deterioration. Here we describe a protocol for developing a new outcome measure using mixed methods to address these limitations.

Methods and Analysis

We will undertake a systematic literature review to identify existing generic, syndrome specific and organ specific definitions for clinically deteriorated, hospitalised adult patients. An international modified Delphi study will generate a short list of candidate definitions. A Nominal Group Technique (NGT), using a trained facilitator, will then take a diverse group of stakeholders through a structured process. The NGT process will be informed by the data generated from the first two stages and will achieve final consensus of a definition for the deteriorated ward patient that is readily extractable from the major EMR platforms.

For peer review only - http://bmjopen.bmj.com/site/about/guidelines.xhtml

Ethics and Dissemination

This study has ethics approval (reference 16399) from the Central Adelaide Local Health Network Human Research Ethics Committee. Results generated from this study will be disseminated through publication and presentation at national and international scientific meetings.

Strengths and Limitations of this study

- This work addresses an important knowledge gap and will assist in developing predictive tools for clinical deterioration.
- The systematic review will be thorough and will scope all relevant available published data to inform the development of the definitions
- The international consensus process will include patients, researchers and clinicians from across different health settings improving the definitions validity
- Determining when to implement augmented organ support varies between individual clinicians and health care settings and bringing the multiple opinions and experiences together into one set of definitions will be challenging.
- The final definition(s) will need to be extractable from the EMR to serve their purpose and will require ongoing refinement and evaluation in large international data sets.

For peer review only

For peer review only - http://bmjopen.bmj.com/site/about/guidelines.xhtml

Introduction

Most patients admitted to hospital recover with treatments that can be administered on the general ward. A small but important group deteriorate however, to the extent that they require augmented organ support **(Figure 1).** [1] In observational studies evaluating this cohort proxy outcomes are used. These include unplanned transfer from the general ward to the Intensive Care Unit (ICU), cardiac arrest and death. [2] The decision to transfer patients to the ICU is dependent on multiple factors, including personalised advance care directives, clinician opinion, local care escalation protocols such as Early Warning Score (EWS) systems, and the availability of ICU resources. [3] Cardiac arrest and death are well-defined and easily measured but are often a very late marker of deterioration. Additionally, cardiac arrest frequency is rare, which limits its use for derivation and validation processes, even in large patient data sets. These factors introduce subjectivity and variability to research that uses these as outcome measures, which in turn hinders the development and accuracy of the increasing numbers of Electronic Medical Record (EMR) linked, algorithmic tools designed to predict clinical Deterioration. [4,5]

Aims

The primary aim of this study is to establish an international consensus definition (or set of syndrome or organ specific definitions) for the deteriorated ward patient. The secondary aim is to do so using data that is commonly available in most EMR's. The definitions will target the time-point that the requirement for augmented organ support first occurs (whilst taking into consideration contextual variables like advanced care directives).

For peer review only - http://bmjopen.bmj.com/site/about/guidelines.xhtml

Methods

Consensus definitions will be established in three stages. Firstly, a systematic literature review will be undertaken to identify existing generic, syndrome and organ specific definitions of clinical deterioration. Secondly, an international modified Delphi study will generate a short list of candidate definitions. Finally, a Nominal Group Technique (NGT) meeting, informed by the data generated from the first two stages, will generate the final definition(s).

Stage 1 - Literature Review

Objective

To identify existing generic, syndrome specific and organ specific definitions for clinically deteriorated, hospitalised, adult patients.

Methods

This systematic review will follow the requirements of Preferred Reporting Items for Systematic Reviews and Meta-analyses Protocol (PRISMA-P). [6]

Phenomenon of interest

Studies that characterise or define deteriorated ward patients. These may be regarding generic (or generalised) deteriorated states associated with the traditional early warning score systems or novel algorithmic, automated deteriorated patient surveillance tools. [7] They may be regarding syndromes specific to clinical deterioration, such as sepsis and associated definitions including Sepsis-3. [8] They may also be regarding specific organ dysfunctions, acute decompensated liver failure being a relevant and common example.

For peer review only

Search Strategy

Studies will be identified using Medical Literature Analysis and Retrieval System Online (MEDLINE), Excerpta Medica database (EMBASE), Cumulative Index to Nursing and Allied Health Literature (CINAHL), the Cochrane Database of Systematic Reviews and the Cochrane Central Register of Controlled Trials (CENTRAL). Additional papers will be sourced from references of included studies, reviews articles and studies from the author libraries.

Search Terms

The following search terms will be included: intensive care, critical care, critical, emergency, deteriorating, deteriorated, definition, electronic patient record, electronic health record, electronic patient record, predictive, unplanned intensive care unit admission, adverse event, terminology, nomenclature, acute, acute care, severe, sudden, rapid response, early warning score, sepsis, septic, shock, shocked, hypoxia, COVID-19, respiratory failure, cardiac failure, liver failure, renal failure, anuria, hypotension, instability, unstable, threshold, acute organ dysfunction and criteria. Additional search terms will be considered after initial trials with the above search terms. A trained medical librarian from the Central Adelaide Local Health Network will assist with the search process.

Study Selection and Data Extraction

Two researchers will independently screen the titles and abstracts of identified studies against the inclusion and exclusion criteria. Disagreement and/or uncertainty regarding study eligibility will be resolved using a third party. Both researchers will independently extract data from included studies using DistillerSR (Evidence Partners, Ottawa, Canada) which will also be used to manage data and identify duplicates.

For peer review only - http://bmjopen.bmj.com/site/about/guidelines.xhtml

Inclusion criteria

Quantitative or qualitative studies published in peer reviewed journals describing definitions of, or criteria specific to, adult (defined as > 16 years of age) clinical deterioration will be eligible for inclusion in this review. Studies published from January 2000 until the day of search completion will be included and no language restrictions will be applied. Google Translate will be used for non-English studies.

Exclusion criteria

Case studies, editorials, grey-literature, letters, practice guidelines and abstract-only reports will be excluded.

Quality Assessment

Different tools will be used to assess the quality of included studies, depending on the type of study. For outcome measure studies the COnsensus-based Standards for the selection of health Measurement INstruments (COSMIN) Risk of Bias checklist will be used. [9] For prediction model studies, the Prediction model Risk Of Bias Assessment Tool (PROBAST) will be used. [10] For qualitative studies the Joanna Briggs Institute Evidence Based Practice Checklist will be used. [11] For clinical guidelines the AGREE II tool will be used. [12] For Randomised Controlled Trials the Cochrane Collaboration Risk of Bias 2 guidelines will be used. [13]

Data Synthesis and analysis

We will generate a list of organ specific, syndrome specific and generic definitions of the deteriorated patient from included studies. These data will be presented in table and text form.

For peer review only

For peer review only - http://bmjopen.bmj.com/site/about/guidelines.xhtml

Stage 2 - Delphi Study

Participants

We aim to include 60 participants in the Delphi study which is consistent with previous Delphi surveys in critical care outcomes and should be adequate to achieve a suitably diverse international sample of stakeholders. [14] Participants will be recruited through the International Society for Rapid Response Systems, the International Forum for Acute Care Trialists and relevant national societies. No formal inclusion criteria will be used however potential participants will be considered based on relevant clinical and research experience, with the aim of ensuring participants are representative of eventual end-users. These will include hospital-based clinical staff working in regional, rural and metropolitan hospitals as well as non-clinician content experts such as researchers and digital health specialists.

Patient and Public Involvement

A small number of health consumer representatives will also be recruited to participate.

Round 1 – Establishing initial definitions (time frame: two months)

Results of the literature review and a list of potential domains, variables and/or parameters will be distributed via email to participants. Participants will provide structured feedback on the merits (or otherwise) of each item. These will then be coalesced into an initial list of potential definitions. Any missed items will be submitted to the process for consideration.

Round 2 – Ranking potential definitions (time frame: two months)

For peer review only - http://bmjopen.bmj.com/site/about/guidelines.xhtml

Participants will rank each potential definition using a 9-point Likert System that is recommended by the Grading of Recommendations, Assessment, Development and Evaluation (GRADE) Working Group Handbook for evaluating outcomes measures. Based on previous work in outcomes research we have defined consensus as 70% of respondents classifying the definitions as 'critical' (score of 7 - 9) and less than 15% determining the definition to be 'not relevant' (score 1 - 3). The results will be aggregated. Any criteria achieving a score of > 70% 'not relevant' will be removed.

Round 3 – Refining aggregated results (time frame: two months)

Aggregated results will be presented to each participant. Definitions that remain, but that have not yet achieved consensus, will be rescored. These results will then be aggregated, and the list finalised. Any definitions that have not achieved consensus after three rounds of scoring will be excluded.

Round 4 – Generating thresholds (time frame: two months)

Participants will propose one threshold for each organ specific definition with an evidence-based justification for the threshold.

Stage 3 - Nominal Group Technique/Consensus meeting (time frame: 1 day)

Nominal Group Technique (NGT) is a validated method for establishing consensus on a specific issue or range of related issues that uses a trained facilitator to take a group of participants through a structured process. [15][16] The NGT meeting will aim to include a diverse range of clinical stakeholders. The target number of participants will be 15 - 20.

For peer review only - http://bmjopen.bmj.com/site/about/guidelines.xhtml

Participants

Participants (both professional and public) will be selected and invited using the same process as described for the Delphi. Participants need not have been involved in the first two stages of the study to take part.

A trained facilitator will lead NGT participants through the structured multi-stage process: Firstly, participants will be presented with an overview of the NGT meeting rationale and aim. Next, participants will be presented with the results of the systematic review and the Delphi process. Participants will then spend 10 - 15 minutes writing a list of bullet point reflections and opinions on the definitions provided, including an opportunity to advocate for additional relevant data not previously included. The facilitator will then get participants to list one reflection/opinion that is yet to be presented. Each original point will be transcribed onto a screen or whiteboard, so all participants can consider and review. This may take several rounds until opinions are exhausted (the aim being to enable all participants to express their views and prevent specific participants having a disproportionate influence).

Participants will then place each definition into two columns: one for inclusion and one for exclusion. The results of this activity will be tabulated and presented. Consensus will be confirmed if more than 70% of participants support its inclusion or exclusion. [16] If there is a lack of consensus on a definition, then the contentious item will be taken back to the group for reappraisal and repeated voting until either consensus or stalemate (two additional voting rounds without consensus) is reached.

For peer review only - http://bmjopen.bmj.com/site/about/guidelines.xhtml

The final stage of the NGT will determine the thresholds (if required) for each of the definitions. Participants will write down opinions/reflections on potential thresholds and these will be collated with each original perspective transcribed. Participants will then provide specific thresholds for relevant definitions; these results will be tabulated, and discussion will be encouraged. The facilitators will present numerous potential thresholds based on the feedback and these will again be voted on. The final set of definition thresholds will be presented to the group and pending agreement, recorded for subsequent publication.

Ethics and Dissemination

This study has ethics approval (reference 16399) from the Central Adelaide Local Health Network Human Research Ethics Committee. Results generated from this study will be disseminated through publication and presentation at national and international scientific meetings.1

Discussion

This protocol describes a three-step consensus building process for developing a definition (or set of definitions) for the deteriorated ward patient. The purpose of this research is to aid the development and improve the performance of automated, EMR linked, digital models that predict clinical deterioration in general ward patients. It will also be useful in evaluations of Early Warning Score and Rapid Response Systems. It is important to note the definition(s) will not be designed as real time decision making adjuncts or to replace complex clinical decision making.

The work has a number of weaknesses. The pandemic has limited the ability of those involved to gather in person (which is the most effective way to build consensus). The increased familiarity with virtual meeting platforms mitigates this to a certain degree. Determining when to implement

For peer review only - http://bmjopen.bmj.com/site/about/guidelines.xhtml

augmented organ support varies between individual clinicians and is influenced by institutional resources and health care settings. Indeed, defining ‘augmented organ support’ is itself fraught with difficulty. It is anticipated bringing the multiple opinions and experiences together into one set of definitions is going to be challenging. Additionally, maintaining consistency across generic, organ specific (i.e., respiratory failure) and syndrome specific (i.e., sepsis) definitions of the deteriorated patient will require discipline and careful planning or risk being of little use in research or clinical practice.

This work has a number of strengths. The specific research question and the methods are novel. The systematic review will be thorough and will ensure all relevant available published data will inform the subsequent modified Delphi survey and nominal group technique, which are the most common and well validated methods for establishing consensus in the medical literature. The definition(s) generated by the study will be evaluated for use as outcome measures when developing predictive tools for clinical deterioration. These may in turn reduce the dependence on the traditional outcome measures, including death, cardiac arrest or unplanned ICU admission, which have specific shortcomings that hinder performance. The definitions will be derived from commonly available EMR data, making them widely applicable as digital health care systems become more widespread. There may be additional uses of the consensus definition(s) beyond the remit of this study, such as comparing acuity between different health care providers and guiding policy. Overall, the published results will (through various means) be relevant to the many thousands of patients annually who clinically deteriorate on hospital wards.

For peer review only - http://bmjopen.bmj.com/site/about/guidelines.xhtml

Trial Status

This is Protocol Version 1 dated 11/02/2022. Recruitment for this study has not begun. It is expected that recruitment for participation in the Delphi and NGT will be completed by July 31st, 2022.

Legend

Figure 1. The schematic representation of potential trajectories for hospitalised patients. Most patients progress along the green line. However, in a small cohort significant deterioration will occur. This may be subject to early intervention or will reach an end point at which they are no longer suitable for management in a ward environment and will be defined a 'deteriorated'

Declarations

Acknowledgments

Nil

Contributors

JM and CA conceptualised the study. JM, CA, OR, CS and DJ undertook the initial methodological planning. GL, PW, DY, SP, AF undertook secondary analysis of the methods and provided updates and corrections. AP and LD provided technical advice. JM and CA wrote the initial manuscript. OR, GL, PW, DY, SP, CS, LD, AP, AF and DJ edited successive drafts and approved the final manuscript.

For peer review only

Funding

PW and OR are both supported by the National Institute for Health and Research Biomedical Research Centre, Oxford. CS does consultancy work for Philips Healthcare.

Data and material

All data and material generated from the study will be published.

Competing interests

None

Patient consent for publication

N/A

Ethics approval

Ethical approval for the Delphi survey and nominal group technique meeting is currently being reviewed by the Central Adelaide Local Health Network Research and Ethics Committee.

Provenance and peer review

Open access

For peer review only

For peer review only - http://bmjopen.bmj.com/site/about/guidelines.xhtml

References

1 Orosz J, Bailey M, Udy A, *et al.* Unplanned ICU Admission from Hospital Wards after Rapid Response Team Review in Australia and New Zealand. *Crit Care Med* 2020;**48**:E550–6.

2 Churpek MM, Yuen TC, Edelson DP. Predicting clinical deterioration in the hospital: The impact of outcome selection. *Resuscitation* 2013;**84**:564–8.

3 Malycha J, Redfern O, Pimentel M, *et al.* Evaluation of a digital system to predict unplanned admissions to the intensive care unit: A mixed-methods approach. *Resusc Plus* 2022;**9**:100193.

4 Escobar GJ, Liu VX, Schuler A, *et al.* Automated Identification of Adults at Risk for In-Hospital Clinical Deterioration. *N Engl J Med* 2020;**383**:1951–60.

5 Churpek MM, Yuen TC, Edelson DP. Risk stratification of hospitalized patients on the wards. *Chest* 2013;**143**:1758–65.

6 Moher D, Liberati A, Tetzlaff J, *et al.* Reprint—Preferred Reporting Items for Systematic Reviews and Meta-Analyses: The PRISMA Statement. 2017;**89**:873–80.

7 Mann KD, Good NM, Fatehi F, *et al.* Predicting Patient Deterioration: A Review of Tools in the Digital Hospital Setting. *J Med Internet Res* 2021;**23**:e28209.

8 Singer M, Deutschman CS, Seymour CW, *et al.* The Third International Consensus Definitions for Sepsis and Septic Shock (Sepsis-3). *JAMA* 2016;**315**:801–10.

9 Mokkink LB, de Vet HCW, Prinsen CAC, *et al.* COSMIN Risk of Bias for systematic reviews of Patient-Reported Outcome Measures. *Qual Life Res* 2018;**27**:1171–9.

10 Wolff RF, Moons KGM, Riley RD, *et al.* PROBAST: A Tool to Assess the Risk of Bias and Applicability of Prediction Model Studies. *Ann Intern Med* 2019;**170**:51–8.

11 Vardell E, Malloy M. Joanna Briggs Institute: an evidence-based practice database. *Med Ref Serv Q* 2013;**32**:434–42.

12 AGREE Collaboration. Development and validation of an international appraisal instrument for assessing the quality of clinical practice guidelines: the AGREE project. *Qual Saf Health Care* 2003;**12**:18–23.

13 Collaborative C. An introduction to Risk of Bias 2. Cochrane. https://methods.cochrane.org/risk-bias-2

14 Sinha IP, Smyth RL, Williamson PR. Using the Delphi technique to determine which outcomes to measure in clinical trials: Recommendations for the future based on a systematic review of existing studies. *PLoS Med* 2011;**8**:e1000393.

For peer review only

For peer review only - http://bmjopen.bmj.com/site/about/guidelines.xhtml

15 Grech C. Use of consensus development to establish national research priorities in critical care. *Aust Crit Care* 2000;**13**:163.

16 Rankin NM, McGregor D, Butow PN, *et al.* Adapting the nominal group technique for priority setting of evidence-practice gaps in implementation science. *BMC Med Res Methodol* 2016;**16**:1–9.

For peer review only

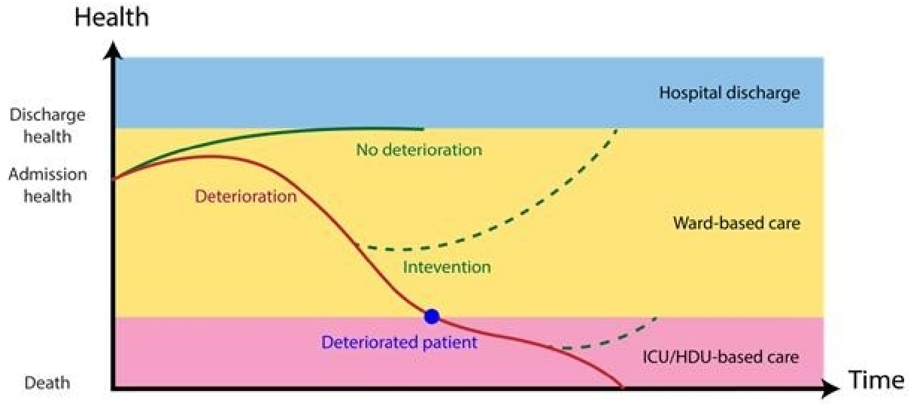

Figure 1. A schematic representation of potential trajectories for hospitalised patients. Most patients progress along the green line. However, in a small cohort, significant deterioration will occur. This deterioration may be subject to early intervention or will reach an end point at which time the patient is no longer suitable for management in a ward environment and will be defined as 'deteriorated'

177x80mm (96 x 96 DPI)

PRISMA-P (Preferred Reporting Items for Systematic review and Meta-Analysis Protocols) 2015 checklist: recommended items to address in a systematic review protocol*

Section and topic	Item No	Checklist item	Comments
**ADMINISTRATIVE INFORMATION**			
Title:			
Identification	1a	Identify the report as a protocol of a systematic review	P1
Update	1b	If the protocol is for an update of a previous systematic review, identify as such	N/A
Registration	2	If registered, provide the name of the registry (such as PROSPERO) and registration number	Not registered
Authors:			
Contact	3a	Provide name, institutional affiliation, e-mail address of all protocol authors; provide physical mailing address of corresponding author	P1-3
Contributions	3b	Describe contributions of protocol authors and identify the guarantor of the review	P15
Amendments	4	If the protocol represents an amendment of a previously completed or published protocol, identify as such and list changes; otherwise, state plan for documenting important protocol amendments	N/A
Support:			
Sources	5a	Indicate sources of financial or other support for the review	P16
Sponsor	5b	Provide name for the review funder and/or sponsor	P16
Role of sponsor or funder	5c	Describe roles of funder(s), sponsor(s), and/or institution(s), if any, in developing the protocol	P16
**INTRODUCTION**			
Rationale	6	Describe the rationale for the review in the context of what is already known	P6
Objectives	7	Provide an explicit statement of the question(s) the review will address with reference to participants, interventions, comparators, and outcomes (PICO)	P6
**METHODS**			
Eligibility criteria	8	Specify the study characteristics (such as PICO, study design, setting, time frame) and report characteristics (such as years considered, language, publication status) to be used as criteria for eligibility for the review	P8-9
Information sources	9	Describe all intended information sources (such as electronic databases, contact with study authors, trial registers or other grey literature sources) with planned dates of coverage	P8-9
Search strategy	10	Present draft of search strategy to be used for at least one electronic database, including planned limits, such that it could be repeated	P8
Study records:			
Data management	11a	Describe the mechanism(s) that will be used to manage records and data throughout the review	P8-9
Selection process	11b	State the process that will be used for selecting studies (such as two independent reviewers) through each phase of the	P8

For peer review only

		review (that is, screening, eligibility and inclusion in meta-analysis)	
Data collection process	11c	Describe planned method of extracting data from reports (such as piloting forms, done independently, in duplicate), any processes for obtaining and confirming data from investigators	P8
Data items	12	List and define all variables for which data will be sought (such as PICO items, funding sources), any pre-planned data assumptions and simplifications	P8
Outcomes and prioritization	13	List and define all outcomes for which data will be sought, including prioritization of main and additional outcomes, with rationale	N/A
Risk of bias in individual studies	14	Describe anticipated methods for assessing risk of bias of individual studies, including whether this will be done at the outcome or study level, or both; state how this information will be used in data synthesis	P9
Data synthesis	15a	Describe criteria under which study data will be quantitatively synthesised	P9
	15b	If data are appropriate for quantitative synthesis, describe planned summary measures, methods of handling data and methods of combining data from studies, including any planned exploration of consistency (such as I^2, Kendall's τ)	N/A
	15c	Describe any proposed additional analyses (such as sensitivity or subgroup analyses, meta-regression)	N/A
	15d	If quantitative synthesis is not appropriate, describe the type of summary planned	P9
Meta-bias(es)	16	Specify any planned assessment of meta-bias(es) (such as publication bias across studies, selective reporting within studies)	N/A
Confidence in cumulative evidence	17	Describe how the strength of the body of evidence will be assessed (such as GRADE)	N/A

*** It is strongly recommended that this checklist be read in conjunction with the PRISMA-P Explanation and Elaboration (cite when available) for important clarification on the items. Amendments to a review protocol should be tracked and dated. The copyright for PRISMA-P (including checklist) is held by the PRISMA-P Group and is distributed under a Creative Commons Attribution Licence 4.0.**

From: Shamseer L, Moher D, Clarke M, Ghersi D, Liberati A, Petticrew M, Shekelle P, Stewart L, PRISMA-P Group. Preferred reporting items for systematic review and meta-analysis protocols (PRISMA-P) 2015: elaboration and explanation. BMJ. 2015 Jan 2;349(jan02 1):g7647.

For peer review only
